# Shorter Migration Distance and Breeding Latitude Correlate With Earlier Egg‐Laying Across the Northeastern Pacific Ocean Range of the Rhinoceros Auklet (*Cerorhinca monocerata*)

**DOI:** 10.1002/ece3.70370

**Published:** 2024-10-09

**Authors:** Cayle J. R. Cross, Katharine R. Studholme, Mark C. Drever, Alice D. Domalik, J. Mark Hipfner, Glenn T. Crossin

**Affiliations:** ^1^ Department of Biology Dalhousie University Halifax Nova Scotia Canada; ^2^ Wildlife Research Division Environment and Climate Change Canada Dartmouth Nova Scotia Canada; ^3^ Wildlife Research Division Environment and Climate Change Canada Delta British Columbia Canada

**Keywords:** *Cerorhinca monocerata*, geolocation, GLS, immersion sensors, migration distance, rhinoceros auklet, timing of breeding

## Abstract

Models of migratory behavior predict trade‐offs between fitness costs and benefits with respect to migration distance. Shorter migration distances may confer a fitness benefit by facilitating earlier breeding, however this is rarely investigated. We tested this hypothesis using a large‐scale geolocation (GLS) dataset from 109 rhinoceros auklets (*Cerorhinca monocerata*), a differentially migrating seabird, that was tagged at 12 breeding colonies along the Pacific Coast of North America, spanning southern California to the eastern Aleutian Islands, Alaska. Using GLS‐based position estimates, we determined the geographic centroid of the pelagic areas occupied by birds in winter (1 January–28 February) and then calculated the distance between their wintering centroids and colony of origin. We then used GLS light‐intensity and salt‐water immersion (wet/dry) data to determine each individual's date of egg‐laying the following spring. Rhinoceros auklets were very widely distributed across the northeastern Pacific Ocean in winter. Among all individuals, the distance between winter centroids and breeding colonies ranged from < 100 to > 2500 km, being greater among individuals originating from colonies at higher latitudes. As predicted, migration distance and colony latitude were positively related to lay date: after accounting for colony‐level differences in phenology, individuals that migrated shorter distances tended to lay their eggs earlier, a pattern that emerged across all populations. Our study links the migration distance of rhinoceros auklets to a fitness‐related outcome, underscoring the selective pressure that migration exerts on subsequent breeding activity.

## Graphical abstract

Using a GLS dataset, we investigated relationships between overwintering migration distances from the breeding colony and egg‐laying date in the subsequent breeding season in the rhinoceros auklet (*Cerorhinca monocerata*). We found evidence to support that shorter migration distances led to earlier lay dates in this species.

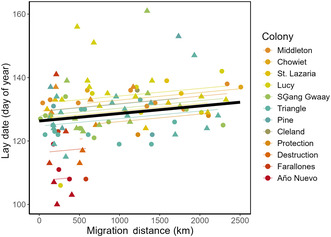

## 1 Background

Migration is a behavior present in nearly all major lineages in the animal kingdom (Boyle 2008; Buchan et al. 2020; Dingle and Drake 2007), and its effects range from individual fitness to ecosystem structure and function (Nathan et al. 2008). At an individual level, the timing, route, and speed of the prebreeding migration determine the date of arrival back to the breeding site, so these factors should have significant fitness consequences (Alerstam 2011; Gienapp and Bregnballe 2012). However, relatively few studies have demonstrated links between variation in migratory behavior and fitness outcomes, even though the distances that individuals migrate and the schedules they keep can vary greatly within and among populations (Chapman et al. 2011; Cristol, Baker, and Carbone 1999; Kentie et al. 2023; Newton 2012).

Differential migration involves the migration of all individuals in a population, but at different scales. Individual differences in migration tactic, such as the distance traveled or the destination selected, are often attributable to some predictable population‐level classification, such as sex or age (Cristol, Baker, and Carbone 1999). Theory suggests that differential migration will evolve within a population when the costs and benefits of long‐ versus short‐distance migrations have comparable fitness outcomes (Chapman et al. 2011; Cristol, Baker, and Carbone 1999). Extending from studies of birds (the most extensively studied taxa), three main, nonexclusive hypotheses have been proposed to explain why individuals or populations might adopt different migration tactics and move to nonbreeding areas located at varying distances from their breeding locale: variation in body size (the body size hypothesis), variation in intraspecific dominance status (the social‐dominance hypothesis), and variation in timing of return to the breeding site (the arrival‐time hypothesis) (Cristol, Baker, and Carbone 1999). Importantly, all three of these models of avian migration assume the existence of some fitness benefit in remaining close to the breeding area. One widely posited benefit of shorter migration is a potentially earlier egg‐laying date. Indeed, the timing of breeding is among the strongest predictors of individual fitness in birds (Perrins 1970; Williams 2012).

The rhinoceros auklet (*Cerorhinca monocerata*) is a widely distributed, burrow‐nesting seabird that lays a single‐egg clutch, and in which both sexes incubate eggs and provision nestlings, visiting the colony only at night. Despite its name, it is more closely aligned with the puffin tribe Fraterculini than the auklet tribe Aethiini (Smith and Clarke 2015). Rhinoceros auklets are long‐lived seabirds, with the oldest recaptured individual being over 31 years old, but more commonly living into their 20s (Hipfner et al. 2019). Like many seabirds (Phillips et al. 2017), rhinoceros auklets are highly migratory, with some traveling thousands of kilometers during the non‐breeding season before returning to natal colonies in the early spring to breed (Hipfner et al. 2020). Of note, this species is a differential migrant as its nonbreeding migration distances vary substantially among individuals, both within and among colonies. Migration distances tend to be shorter from colonies toward the southern end of their range and greater from colonies further north (Hipfner et al. 2020). Ultimately, as in most birds, rhinoceros auklets that lay their eggs earlier in the season tend to have higher reproductive success than those that lay later (see Verhulst and Nilsson 2008), due largely to the matching of breeding activity to periods of high food production (Watanuki et al. 2009; Wilson and Manuwal 1986).

We used a large geolocation (GLS) dataset consisting of tracks from 109 rhinoceros auklets tagged at 12 northeastern Pacific Ocean breeding colonies extending from southern California to the Aleutian Islands, Alaska. GLS data from these same colonies formed the basis for a previous study examining drivers of genetic structure in rhinoceros auklets (Hipfner et al. 2020). We calculated the geographic centroid of the at‐sea area occupied by each individual during the nonbreeding period in winter (1 January–28 February) and then calculated the distance between each bird's wintering centroid and its breeding colony. We then used a combination of GLS‐recorded light‐intensity and salt‐water immersion (wet/dry) data to determine the dates that individuals laid their eggs. Recent studies have used GLS data in this manner to determine the phenology of burrow nesting (Gaston, Hashimoto, and Wilson 2017), cavity nesting (Gow, Wiebe, and Fox 2015; Gow 2016; Schaub et al. 2019), and ground nesting birds (Burger et al. 2012). We hypothesize that migration distance from wintering areas to breeding colonies will affect the breeding phenology of rhinoceros auklets, and we predict that, in addition to an expected correlation between lay date and breeding latitude, a positive relationship should occur between migration distance and the date of egg‐laying (i.e., birds that travel further nest later in the season).

## 2 Methods and Materials

### 2.1 Study Sites, Years, and Sample Sizes

Light‐level geolocation tags (GLS; Intigeo‐C65, Migrate Technology Ltd., 1.0 g, range 4, mode 6) were deployed on rhinoceros auklets late in the 2013 and 2014 breeding seasons and recovered when birds returned to breed in the springs of 2014 and 2015. Four auklets were recaptured 2 years after they were tagged, thereby capturing two full migration cycles, but we restricted our analyses to the first year of data. Deployments occurred on three colonies in Alaska, USA (Chowiet, Middleton, and St. Lazaria islands); five colonies in British Columbia, Canada, (Lucy, Pine, Triangle, and Cleland islands, and SGang Gwaay); two colonies in Washington State, USA (Protection and Destruction islands); and two colonies in California, USA (Southeast Farallon and Año Nuevo islands). The numbers and sexes of birds tagged are shown in Table 1. Sex was determined from bill measurements (Pyle 2008) and confirmed with genetic sexing whenever possible (Hipfner et al. 2020).

**TABLE 1:** Details related to the recoveries of geolocation tags deployed on rhinoceros auklets (*Cerorhinca monocerata*) on 12 breeding colonies in the northeastern Pacific Ocean.

Colony	Province/State	Year of tracking	No. of females	No. of males	Total	Range of migration distances (km)
Middleton	AK	2014–15	5	8	13	43–2339
St. Lazaria	AK	2014–15	5	3	8	98–2097
Chowiet	AK	2014–15	0	2	2	598–2508
Lucy	BC	2014–15	5	3	8	472–2273
		2015–16	6	3	9	265–2324
SGang Gwaay	BC	2014–15	4	3	7	139–1752
		2015–16	6	7	13	14–2288
Triangle	BC	2014–15	12	0	12	124–2226
		2015–16	4	10	14	73–1931
Pine	BC	2014–15	1	1	2	171–1734
		2015–16	2	2	4	141–1406
Cleland	BC	2014–15	0	1	1	1220
Protection	WA	2015–16	1	2	3	704–2106
Destruction	WA	2015–16	2	0	2	503–538
Farallones	CA	2014–15	3	2	5	127–580
Año Nuevo	CA	2014–15	3	3	6	176–405
Total			59	50	109	

*Note:* The interindividual range of migration distances (i.e., the distance between the winter distribution centroid to the breeding colony) within each colony is also presented. Colonies are ordered latitudinally from north to south.

### 2.2 Determining Migration Distance Between Winter Centroids and Breeding Colonies

The GLS dataset consisting of tracks from 109 rhinoceros auklets breeding on 12 North Pacific colonies was published previously (Hipfner et al. 2020). Colony coordinates and breeding population sizes, as well as detailed methods, can be found there. Briefly, the tracks were derived from GLS light records using the R package *TwilightFree* (Bindoff et al. 2018), and consisted of geolocations based on a 1° × 1° grid with one geolocation per day from tag deployment to retrieval (Hipfner et al. 2020). For information regarding the potential effect of geolocation uncertainty in our results, refer to Appendix 1.

**FIGURE 1:**
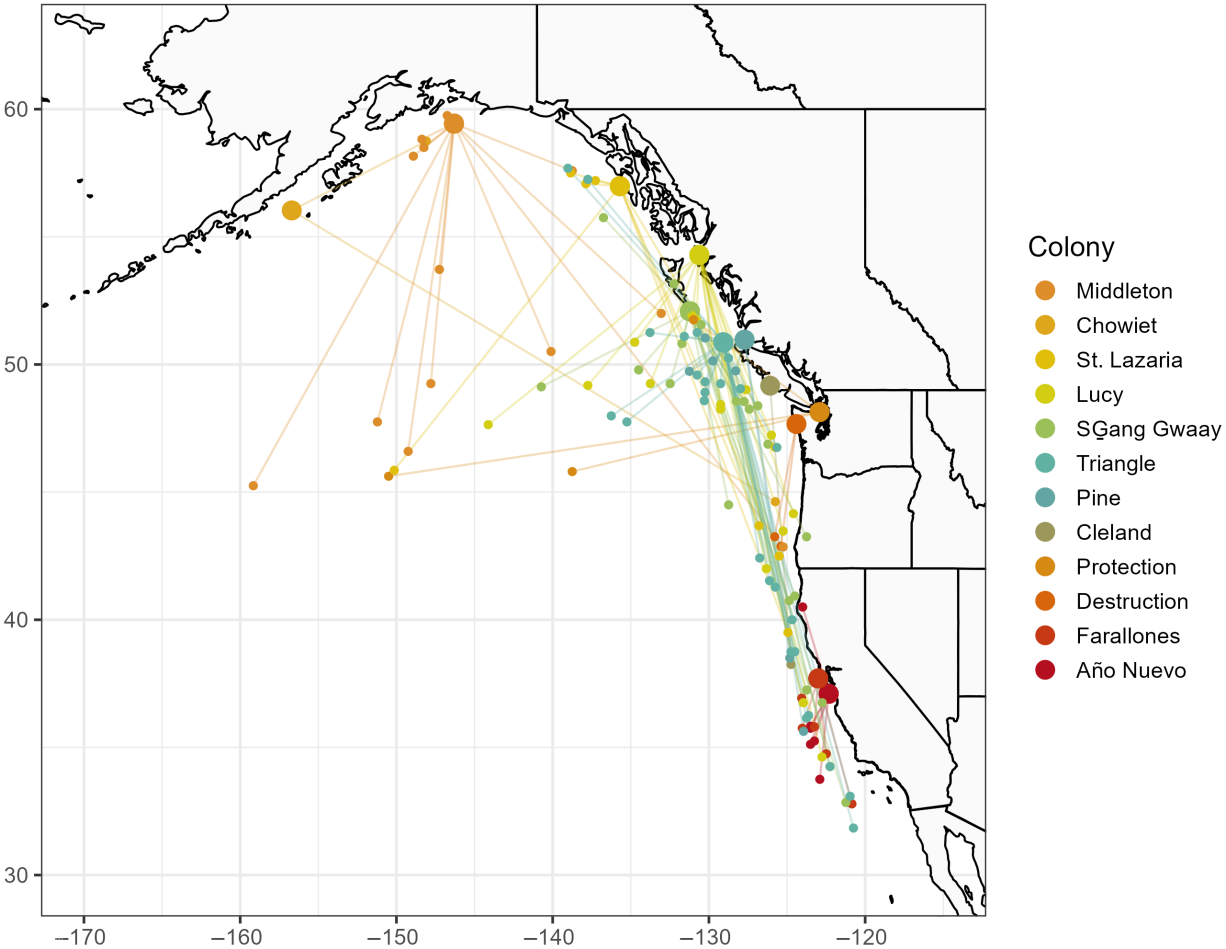
The 90% winter utilization distribution centroids of 109 rhinoceros auklets (*Cerorhinca monocerata*) from 12 colonies spanning the breeding range in the northeast Pacific Ocean.

Using the geolocations from 1 January to 28 February, we determined each individual's at‐sea 90% habitat utilization distribution centroid (UD) using the “kernelUD” function in package *adehabitat* in R (Calenge 2006; Hipfner et al. 2020). Using the centroids of these 90% UDs, we calculated “migration distance” as the distance from the individual's breeding colony to its winter distribution centroid using the “distHaversine” function in package *geosphere* (Hijmans et al. 2019; Figure 1). The latitude of each 90% UD centroid was also recorded, which we termed the “wintering latitude.” Across all birds, wintering latitude was correlated with wintering longitude (Pearson's correlation coefficient *r* = −0.69, *p* < 0.001, *N* = 109), such that birds that were located further southward during the winter period tended to be found to be further eastward (Figure 1). Additionally, birds breeding at the most northerly colonies tended to have overwintering centroids that were also more northerly, while southern colonies had more southerly centroids. In other words, there was a direct relationship between colony latitude and overwintering centroid latitude (*r* = 0.55, *p* ≤ 0.001).

### 2.3 Estimating Egg‐Laying Dates

Field observations of actual laying dates were only possible for ~20% of the birds in this study, all from the British Columbia colonies. These laying dates were determined as part of a collaborative study investigating relationships between overwinter location and the deposition of contaminants in eggs (Elliott et al. 2021)—itself part of a broader, long‐term Environment and Climate Change Canada program on marine contaminants (Elliot et al. 1989). For the remaining ~80% of birds, we estimated laying dates using both the GLS light waveform data (light intensity through time) and the immersion data (wet/dry) inferred from conductivity sensors embedded in the GLS loggers. If a GLS logger detected no light during known daylight hours, or extended “dry” periods outside the migratory period, we inferred that the GLS‐bearing bird was in its nesting burrow at that time.

To determine egg‐laying date from light waveforms, we first used the “twilightCalc” function in the R package *GeoLight* (Lisovski and Hahn 2012) to identify daily sunrises and sunsets at a threshold of 3 lx. Since false sunrise and sunset events can occur due to shading of the tag during the day or, rarely, artificial light interference at night, any sunrise or sunset events that occurred < 5 h apart (minimum day/night length for 60° N) were flagged. These flagged events were then visually inspected and removed from the dataset if they did not align with expected sunrise and sunset times as predicted by the trend in previous sunrise and sunset events. Once a series of reasonable sunrise and sunset events were established, daylight periods spent in the burrow were identified when no sunrise occurred in the 24 h following a sunset. This method cannot determine whether a bird leaves and re‐enters a burrow during the night.

The immersion sensors on the loggers sampled conductivity every 30 s, and then recorded the number of “wet” events occurring in every 10‐min period, with values ranging from 0 to 20 (i.e., 0 = no wet events, 20 = maximum wet events). During the nonbreeding period, when not actively migrating, rhinoceros auklets spend almost all their time on the water; therefore, only short periods of “dry” would be expected during this time, corresponding to bouts of flight between foraging areas. While breeding, the auklets are “centrally placed” and burrow‐oriented (*sensu* Orians and Pearson 1979), therefore breeding activity can be inferred from extended periods of dryness occurring over consecutive nights and days. We therefore defined periods of burrow occupancy as any period where the tag remained dry for five or more hours during the breeding season.

**FIGURE 2:**
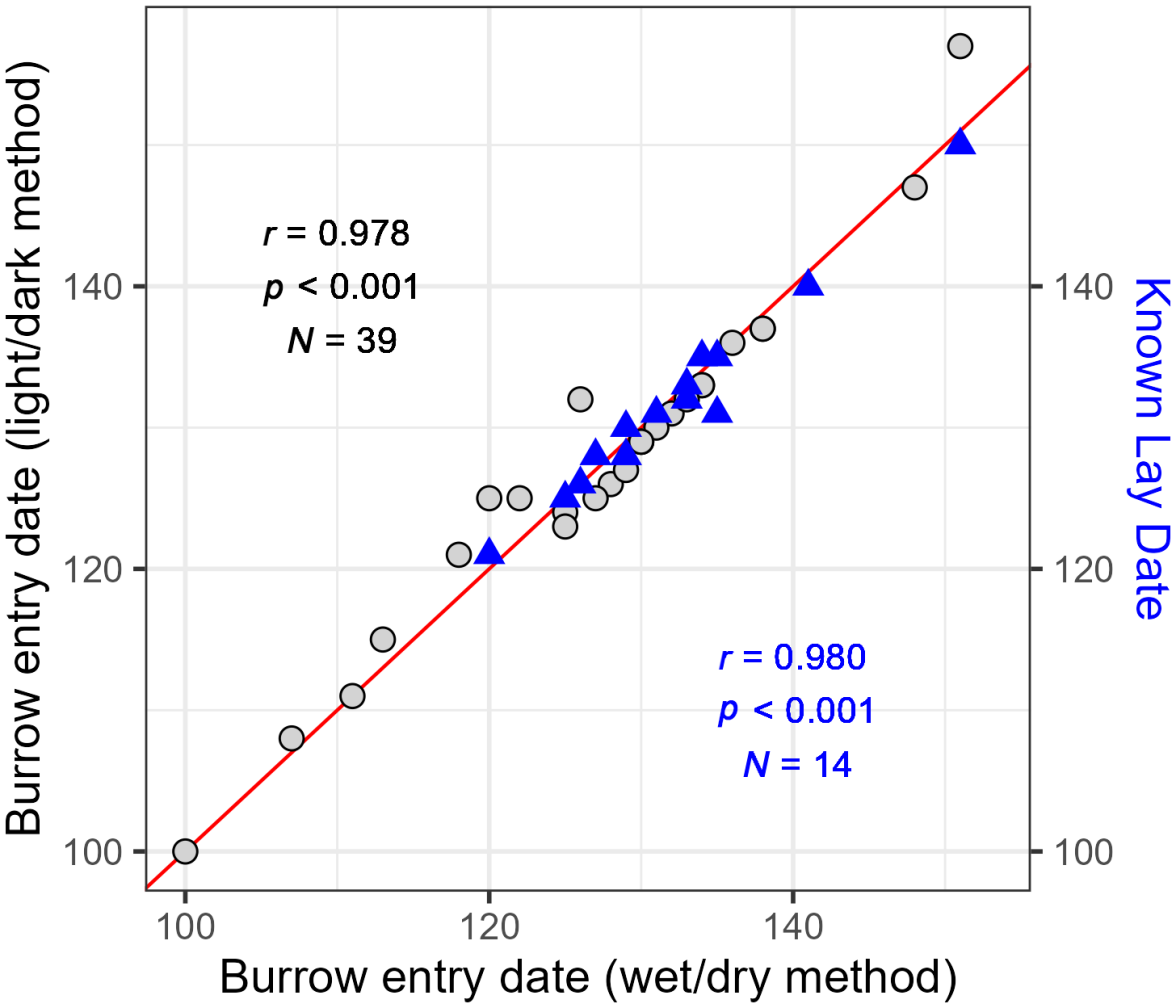
Correlation (gray points, black line) between the light/dark geolocation method and the wet/dry sensor data method for determining dates of breeding burrow re‐occupancy by female rhinoceros auklets (*Cerorhinca monocerata*). Also shown is the correlation between the wet/dry sensor method of breeding burrow re‐occupancy and actual, known laying dates for the same individual females (blue triangles, blue line). The red line indicates a 1:1 relationship. All dates are noted as day of year (January 1 is DOY = 1).

In combination, the light and immersion data allowed us to determine when incubation started for each tagged bird, and there was a strong positive relationship between these two methods (Figure 2). Both members of a breeding pair are usually present in the burrow when the egg is laid, and males typically take the first incubation shift (Kubo et al. 2018). Incubation shifts typically last ~24 h and exchanges of duty occur only at night (Wilson and Manuwal 1986); therefore, we subtracted 1 day from the observed start of incubation by tagged females to back‐calculate their laying dates. We assumed a priori that males start incubating when their mates lay the egg. Additional details concerning the processing of GLS waveforms and immersion data to estimate laying date can be found in Appendix 2.

### 2.4 Data Analyses

First, to test whether males and females differ in migration distance between their 90% UD winter distribution centroid and their breeding colony, we fit a linear mixed model (type III sums of squares) using the *lme4* package in R (Bates et al. 2015):

$$\text{Migration distance}\sim \mathrm{Sex}+\text{Year}+\text{Wintering latitude}+\left(1|\text{Colony}\right)$$

In this model, Sex was a parameter that indicated the average difference in migration distance between sexes (treated as a categorical variable), Year was the parameter that tested for differences in migration distance between 2014 and 2015 (treated as a categorical variable), and wintering latitude was a parameter that denoted the slope of the relationship with the latitudinal center of the 90% UD winter centroid (a continuous variable, scaled by subtracting the mean and dividing by the standard deviation). Colony was treated as a random effect both to address unbalanced design (uneven numbers of birds and sexes were tagged across colonies and years; Table 1), and to allow us to compare variation in migration distance among colonies and among birds within the same colony. All two‐way interaction terms were included in the initial model but were non‐significant (all *p* > 0.200; type III sums of squares). Interactions were therefore removed and we report only the main effects (Table 2).

**TABLE 2:** Summary of a linear mixed‐effects model examining the influence of fixed and random effects on migration distance (measured as the distances between wintering distribution centroids and breeding colonies) for 109 rhinoceros auklets (*Cerorhinca monocerata*).

Fixed effects	Estimate	Standard error	DF	*t*‐ratio	*p*
Intercept*	766.2	253.4	11.4	3.02	0.011
Year (2015)	40.3	67.1	96.1	0.60	0.550
Sex (F)	12.6	55.1	94.8	0.23	0.820
Wintering latitude (scaled)*	−708.0	31.6	95.5	−22.43	< 0.001

Random effect	Standard deviation				
Colony	855.5				
Total	266.6				

*Note:* Wintering latitude is the latitude of the 90% utilization distribution centroid of auklets during late winter (1 January–28 February) and was scaled to its mean and standard deviation. Statistically significant effects (*α* = 0.05) are indicated by an asterisk *.

Second, to test our prediction that a shorter migration distance between an individual's wintering distribution and its breeding colony leads to an earlier date of egg‐laying, we ran a second linear mixed model (type III sums of squares):

$$\begin{array}{c} \text{Estimated laying date}\sim \text{Year}+\mathrm{Sex}+\text{Colony latitude}+\\ \text{Migration distance}+\left(1|\text{Colony}\right) \end{array}$$

Although it may seem nonintuitive to include sex in a model exploring laying date given only females lay eggs, we include sex because we assumed a priori that males start incubating when their mates lay the egg, that is, that the male of the pair takes the first incubation shift (Kubo et al. 2018; Wilson and Manuwal 1986). This model allows for variation in mean lay date among colonies and assumes that the relationship (slope) between lay date and migration distance is the same for all birds, regardless of colony of origin. To test this assumption, we compared this model to one where these relationships could vary by colony by including a random slope term for migration distance by colony (migration distance|colony). A Likelihood Ratio Test (LRT) comparing the two models indicated the random slope did not result in a significant improvement in model fit (LRT: χ^2^ = 1.67, df = 2, *p* = 0.43), thus we retained the intercept‐only model with no random slopes.

Residuals of the first and second models were examined for homoscedasticity and normality. Shapiro–Wilks tests indicated that residuals were normally distributed in both models (*p* > 0.001). Mixed models were fit with restricted maximum likelihood estimation (REML). All models were run in R version 4.1.0 (R Core Team 2021). To understand our rationale for using linear mixed models over individual general linear models please refer to Appendix 3.

## 3 Results

**FIGURE 3:**
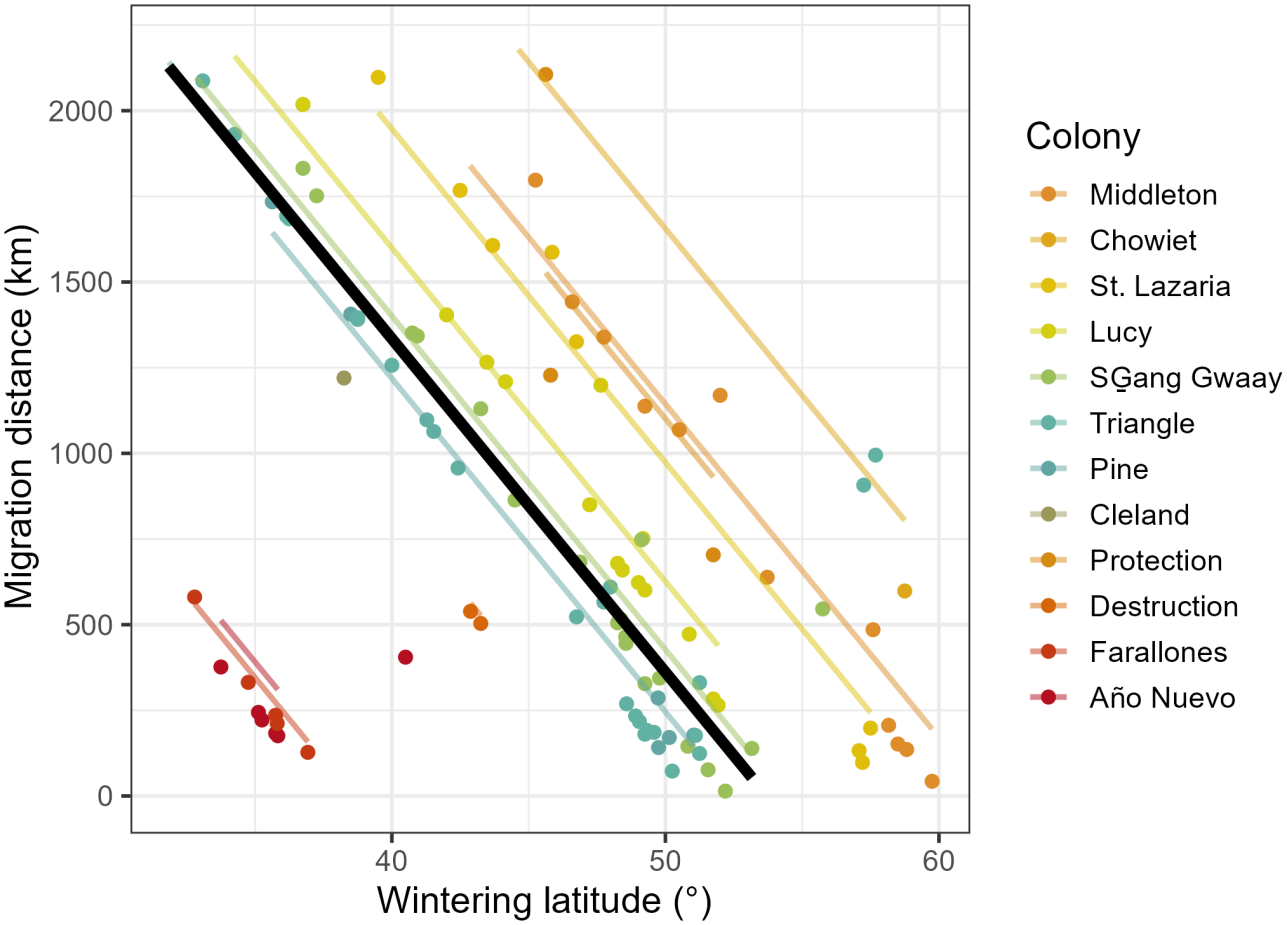
Relationship between the wintering latitude (latitude of the 90% utilization distribution centroid during the winter, 1 January–28 February) and migration distance for 109 rhinoceros auklets (*Cerorhinca monocerata*) breeding on 12 colonies. Colonies are arranged from top to bottom by decreasing latitude (north to south).

During winter (1 January–28 February), rhinoceros auklets were widely distributed across the northeastern Pacific Ocean, from Baja California to the northern Gulf of Alaska, most in waters over the continental shelf and a few birds in deeper waters offshore (Figure 1). The migration distance between the wintering centroids of individuals and their breeding colonies varied widely within and among the 12 colonies we investigated (Figure 3). Migration distance was strongly negatively correlated with wintering latitude (Table 2), indicating that birds that migrated the furthest went further south. Additionally, individuals breeding on southern colonies spent winter closer to their breeding colonies (Figure 3). Migration distances did not vary between years or between males and females (Table 2). Standard deviations of random effects indicated that variance in migration distance among colonies (SD = 856 km) was greater than within colonies (SD = 267 km).

**FIGURE 4:**
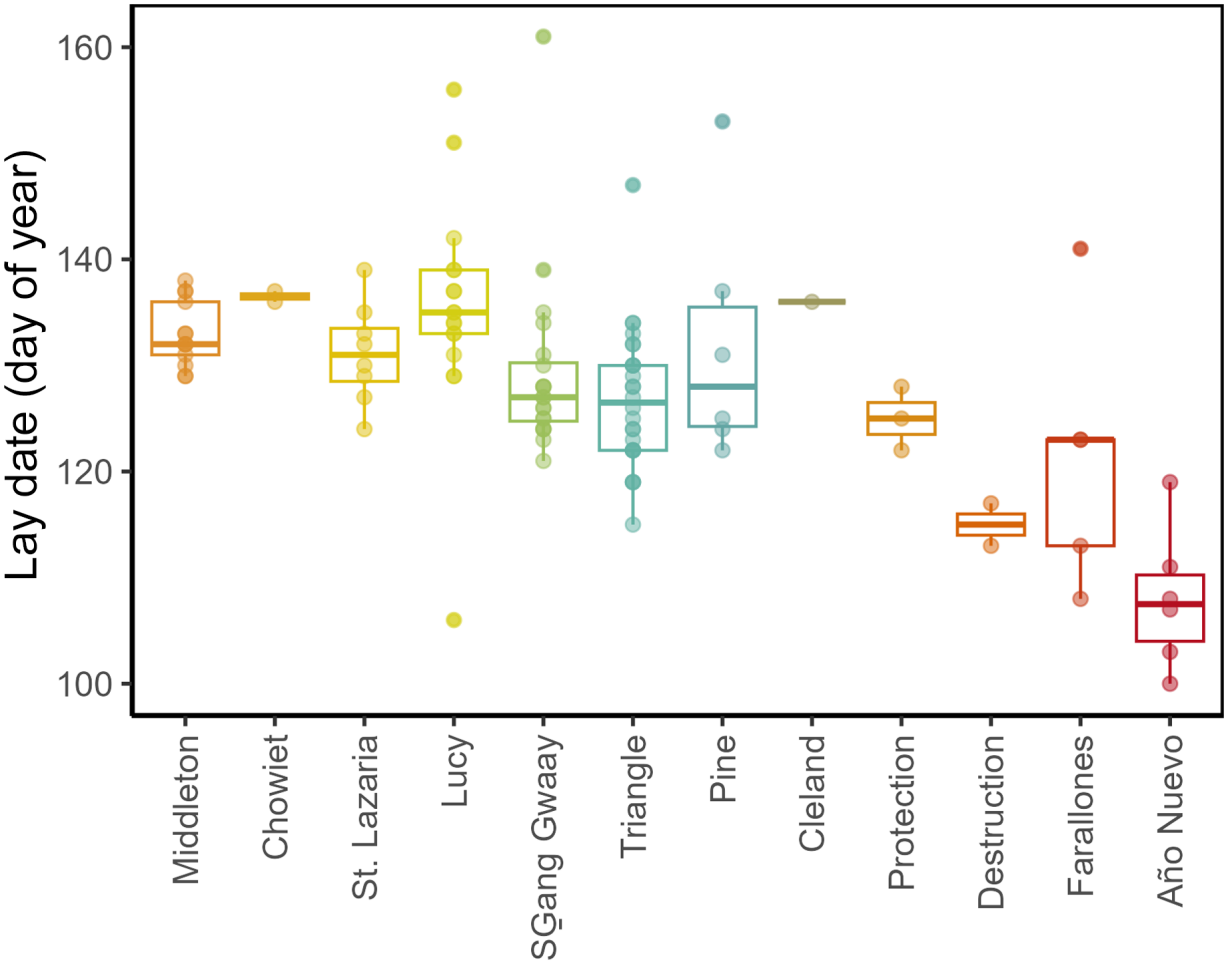
Variation in laying dates of 109 rhinoceros auklets (*Cerorhinca monocerata*) at 12 breeding colonies, as inferred from light‐level geolocation tags. Lay date is noted as day of year (January 1 is DOY = 1). Colonies are ordered from left to right by decreasing latitude.

**FIGURE 5:**
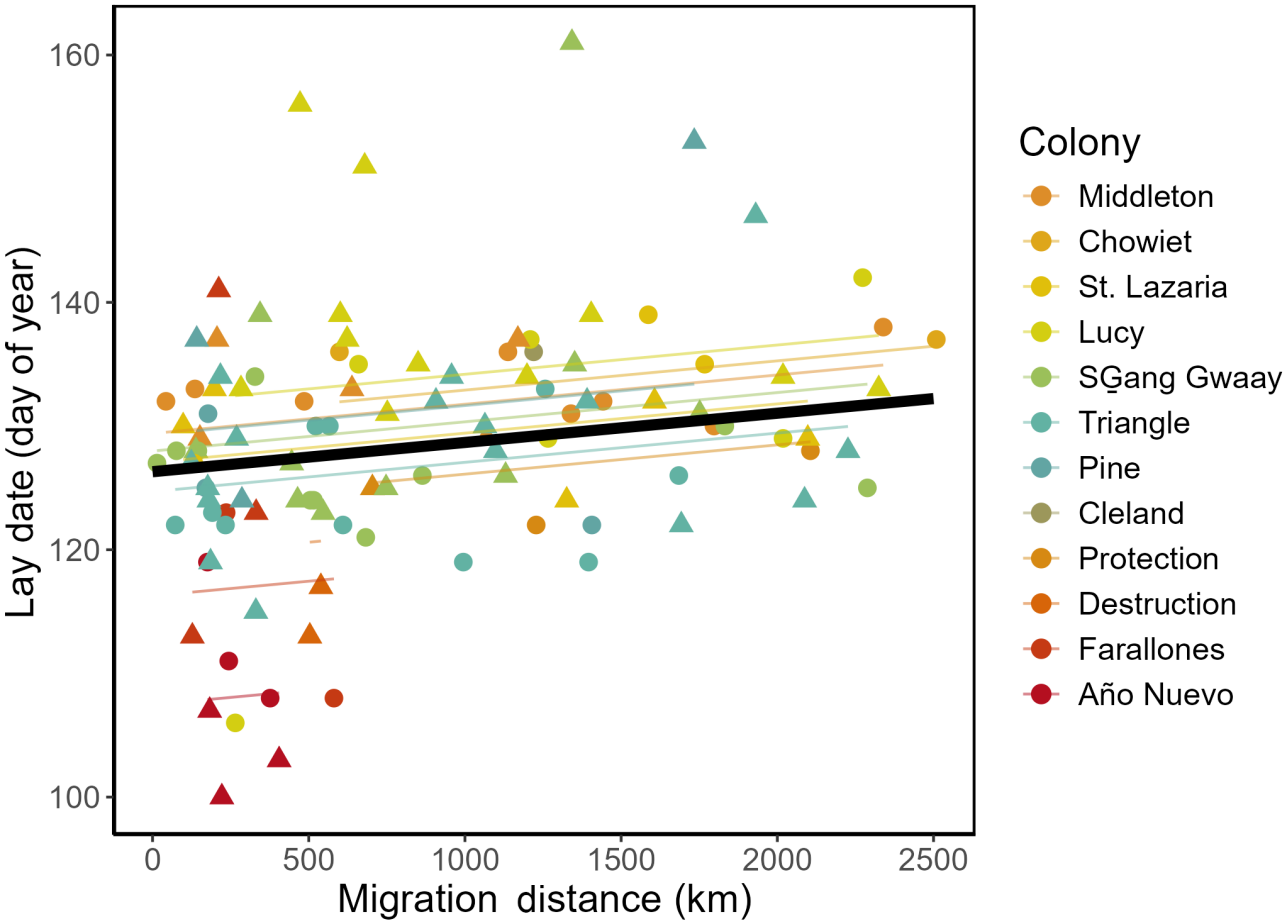
Relationship between the migration distance (distance between the breeding colony and the 90% utilization distribution centroid during the winter, 1 January–28 February) and laying date for 109 rhinoceros auklets (*Cerorhinca monocerata*) breeding on 12 colonies. Triangular points represent female auklets, while circular points represent male auklets. Colonies are arranged from top to bottom by decreasing latitude (north to south). Colored lines indicate predicted values for each colony after averaging between years and sexes, and the solid black line represents the relationship between lay date and migration distance across all colonies. Lay date is noted as day of year (January 1 is DOY = 1).

Laying date varied widely among the12 breeding colonies from California and Washington to British Columbia and Alaska, with the earliest average day dates occurring in the four southernmost colonies (Figure 4). Overall, there was a significant effect of year across birds from all the colonies, indicating that in 2015 birds on average laid eggs 5.1 days earlier relative to 2014 (Table 3). Lay date was strongly correlated with latitude of breeding colony, such that birds from northern breeding colonies had later lay dates, and as indicated by the standardized regression coefficients this effect was stronger than the effect of individual migration distance (Table 3 and Figure 5). As predicted, at the individual level, there was a significant positive relationship between laying date and the migration distance between wintering centroids and breeding colonies: across all 12 colonies, individuals migrating shorter distances laid earlier (Table 3 and Figure 5). The random effect of colony (SD = 4.2 days) was smaller in magnitude than the individual (residual) variance (SD = 7.3 days) (Table 3). In other words, some inherent traits among individual rhinoceros auklets explained nearly the same amount of the variance in laying date as did colony of origin, after accounting for effects of year, sex, colony latitude, and geographic dispersion (latitude, distance) in the winter.

**TABLE 3:** Summary of a linear mixed‐effects model examining laying dates of rhinoceros auklets (*Cerorhinca monocerata*) as a function of year, sex, wintering latitude, and migration distance.

Fixed effects	Estimate	Standard error	DF	*t*‐ratio	*p*
Intercept	129.3	1.8	15.2	68.99	< 0.001
Year (2015)*	−5.1	1.7	98.4	−3.02	0.003
Sex (F)	2.5	1.5	100.6	1.69	0.093
Migration distance (scaled)*	1.6	0.8	98.5	2.15	0.033
Colony latitude (scaled)*	5.2	1.3	8.8	3.97	0.003

Random effect	Standard deviation				
Colony	4.2				
Residual	7.3				

*Note:* Wintering latitude is the latitude of the 90% utilization distribution centroid of auklets during late winter (1 January–28 February), while migration distance represents the distance between this centroid and the breeding colony. Statistically significant effects (*α* = 0.05) are indicated by an asterisk *.

## 4 Discussion

### 4.1 Migration Behavior and Timing of Breeding

Here, we tested the hypothesis that migration distances and the latitude of breeding colonies affect the lay dates of a migratory seabird species breeding at multiple island colonies throughout the Northeast Pacific. Specifically, we predicted that individuals that migrate shorter distances would lay earlier, after accounting for the effect of breeding latitude. To do this, we used a large‐scale GLS dataset from 109 individual rhinoceros auklets, from 12 populations breeding on islands extending from southern California to the eastern Aleutian Islands. In winter, tagged rhinoceros auklets were widely distributed across the northeastern Pacific Ocean, mainly in near‐shore waters over the continental shelf, but some in deeper, more pelagic waters. Among all individuals, the distance between winter centroids and breeding colonies (i.e., the migration distance) ranged from < 100 to > 2500 km. Individuals dispersing from colonies in the northwest parts of the study area traveled further to wintering areas than those dispersing from colonies in the southeast. Migration distances within individual colonies were also widely variable, consistent with the definition of differential migration (Cristol, Baker, and Carbone 1999). Migration distances did not differ significantly between the sexes, which corroborates a similar finding from a study of rhinoceros auklets migrating from a colony in the western Pacific (Teuri Island, Japan; Takahashi et al. 2015).

However, laying dates also varied with colony latitude, being ~10 days earlier at the four southernmost colonies than the most northerly colony (Figure 4). We found that colony latitude had a significant positive relationship with lay date, and results showed a stronger effect than individual variation. The concept of increasing latitude having an impact on lay date in well established in birds (Baker 1939; Burr et al. 2016; Neufeld et al. 2021; Sigh et al. 2021; Wanless et al. 2008), which can be influenced by various local environmental factors (Niehaus and Ydenberg 2006; Votier et al. 2009). At one of our colonies, Triangle Island, the start of egg‐laying by rhinoceros auklets varied by up to 2 weeks interannually and was timed to a critical environmental cue: the marine chlorophyll‐*a* concentration in early spring that predicts annual phenology in the local ecosystem (Crossin et al. 2022). In other seabird species, marine chlorophyll‐*a* concentration can also influence egg laying, specifically in northern populations (Wolf et al. 2009). It may be that individuals that migrate shorter distances are better able to attune themselves to the local breeding environment and consequently to lay their eggs at the best time relative to local food availability (Both and Visser 2001). A broader analysis that links movement patterns and spatiotemporal variability, spring phenology, or prey distribution and abundance in rhinoceros auklets may reveal a putative mechanism linking migration distances and laying date.

Similar to our findings for migration distance and colony latitude, we found that lay date also varied widely among individuals. As predicted, there was a positive relationship between migration distance and laying date; in other words, across all colonies, while accounting for colony‐level differences in phenology, individuals that migrated shorter distances tended to lay their eggs earlier in the following spring. This result supports a key prediction of most models of avian migration behavior: that there is a fitness benefit to migrating shorter distances (Chapman et al. 2011; Cristol, Baker, and Carbone 1999).

In long‐lived seabirds such as rhinoceros auklets (Morrison et al. 2011), high‐quality phenotypes (often older and more experienced birds) typically lay the earliest (De Forest and Gaston 1996; Pyle, Sydeman, and Hester 2001), and we found that individual‐level variation in lay date was greater in magnitude than variation among colonies (Table 3). We therefore propose that a link between phenotypic quality and migration distance exists, such that the most capable individuals migrate shorter distances, enduring the physiological costs associated with a winter spent in the colder, more severe northerly environments (Richman and Lovvorn 2011; Pelletier et al. 2020). Remaining near the colony would then position the birds within the “environment of selection” where they can time egg production based on local indicators of the timing of future food production (Visser et al. 2010; Crossin et al. 2022). This suggests a trade‐off between short‐distance and long‐distance migration tactics, which has been speculated to underlie reproductive success in other seabird species (Garthe et al. 2012) and even in other marine taxa (Farber et al. 2018). The idea that an individual's physiological state or some other measure of its phenotypic quality (Bogdanova et al. 2011; Fayet et al. 2016), or resource availability (Takahashi et al. 2015; Léandri‐Breton et al. 2021), can heavily influence the scope and extent of migration is well established (Chapman et al. 2011; Newton 2012).

Most avian studies have found that individuals that remain resident on their breeding grounds experience a fitness benefit, such as higher survival, over individuals that migrate (Buchan et al. 2020). While our results appear consistent with these findings, the results for other seabirds have been mixed. In northern gannets (*Morus bassanas*), individuals from a North Atlantic breeding colony that migrated to the Gulf of Mexico had higher costs of migration and lower daily energy expenditures in winter compared to individuals that remained further north. However, their survival, arrival time, and hatching success did not differ in relation to migration distance (Pelletier et al. 2020). Similarly, in the little auk (*Alle alle*), a comparison of migration distances differing by twofold found no apparent impact on the timing or success of reproduction (Dufour et al. 2021). These studies demonstrate that in some seabirds, the putative trade‐off of migrating further distances from breeding colonies is not consequential.

In contrast, a study of European shags (*Phalacrocorax aristotelis*) found that pairs remaining around their colony during the wintering period had hatch dates ~12 days earlier than pairs where one or both mates undertook more distant, seaward migrations (Grist et al. 2017). Similarly, sedentary female wandering albatrosses (*Diomedea exulans*) were capable of breeding in consecutive years as opposed to migratory females which bred biennially (which is the primary tactic) (Weimerskirch et al. 2015). Finally, in an expansive multicolony study of Atlantic puffins (*Fratercula arctica*) (Fayet et al. 2017), a close relative of the rhinoceros auklet (Smith and Clarke 2015), the interindividual variation in migration distances across 13 study colonies was comparable to that found in this study, and breeding colony productivity declined with increasing migration distance, increasing population size, and increasing winter latitude. These latter studies emphasize that remaining closer to the breeding colony can result in a fitness benefit. Ultimately, the balance of costs and benefits associated with different migration distances or sedentary behavior depends much on the species in question and its biogeography. Our results for rhinoceros auklets align with those of other seabirds, which suggests a fitness benefit for remaining closer to the breeding colony during the winter.

## 5 Conclusion

Results of this study of the rhinoceros auklet, an abundant and widely distributed North Pacific seabird, add to a rapidly growing body of evidence indicating that variation in migratory behavior can have significant fitness‐related consequences. We believe that our large‐scale, multi‐colony approach provides a productive path forward as we seek to better understand the drivers of migration behavior. More research is needed into the mechanistic (physiological, behavioral) causes and consequences of variation in migration behavior, both among colonies and individuals, especially considering ongoing rapid environmental change. Additionally, previous work has shown that rhinoceros auklets from the northeast and northwest Pacific Ocean exhibit high genetic differentiation, and that individuals from these regions do not mix (Hipfner et al. 2020). Given the relative isolation between east and west, it would be informative to examine whether the links between migration distance and fitness metrics shown in this study (northeast Pacific) have also evolved in the northwest Pacific (see Hipfner et al. 2020; Takahashi et al. 2015).

## Author Contributions

**Cayle J. R. Cross:** conceptualization (supporting), formal analysis (equal), methodology (supporting), writing – original draft (lead), writing – review and editing (equal). **Katharine R. Studholme:** formal analysis (equal), methodology (equal), writing – review and editing (equal). **Mark C. Drever:** conceptualization (supporting), formal analysis (lead), methodology (equal), writing – review and editing (equal). **Alice D. Domalik:** data curation (equal), formal analysis (equal), writing – review and editing (equal). **J. Mark Hipfner:** conceptualization (equal), funding acquisition (lead), supervision (supporting), visualization (equal), writing – review and editing (equal). **Glenn T. Crossin:** conceptualization (equal), funding acquisition (lead), methodology (equal), supervision (lead), writing – original draft (supporting), writing – review and editing (equal).

## Ethics Statement

Fieldwork on the Triangle Island ecological reserve was approved by British Columbia Parks, the Tlatlasikwala First Nations, and the Quatsino First Nations (BC Parks: 102237). All wildlife sampling protocols were approved by Simon Fraser University Animal Care Services (2010–2014: 974B‐94) and Environment Canada's Western and Northern Animal Care Committee (2015–2017: 15MH01, 16MH01, 17MH01). Migratory birds scientific permits included BC‐10‐0017, BC‐11‐0016, BC‐13‐0018, BC‐14‐0026#1, BC‐15‐0005, BC‐16‐0012 and BC‐17‐0028. The banding permit for all years was 10667F. We thank the Bird Banding Laboratory of the United States Geological Survey, the United States Fish and Wildlife Service, the Alaska Maritime National Wildlife Refuge, British Columbia Parks, and Environment and Climate Change Canada for permits to conduct the research.

## Conflicts of Interest

The authors declare no conflicts of interest.

## Figures and Tables

**FIGURE A1:**
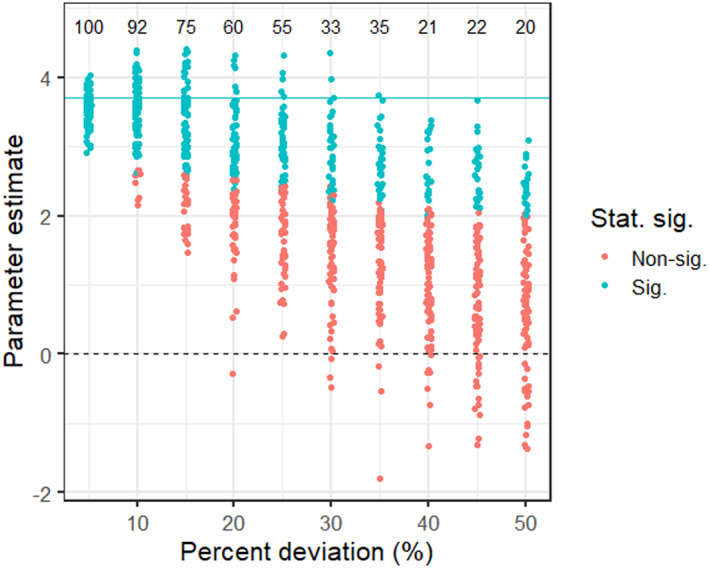
Post hoc analyses examining the sensitivity of the correlation between laying date migration distance (distance between the breeding colony and the 90% utilization distribution centroid during the winter, 1 January–28 February), based on 100 permutations adding additional noise to the distance values. Parameter estimates are derived from the GLMM and their values indicate the strength and direction of the correlation (positive or negative). The color indicates statistical significance at *α* = 0.05. The numbers at the top of the figure indicate the percent of the simulations with statistically significant results.

**FIGURE B1:**
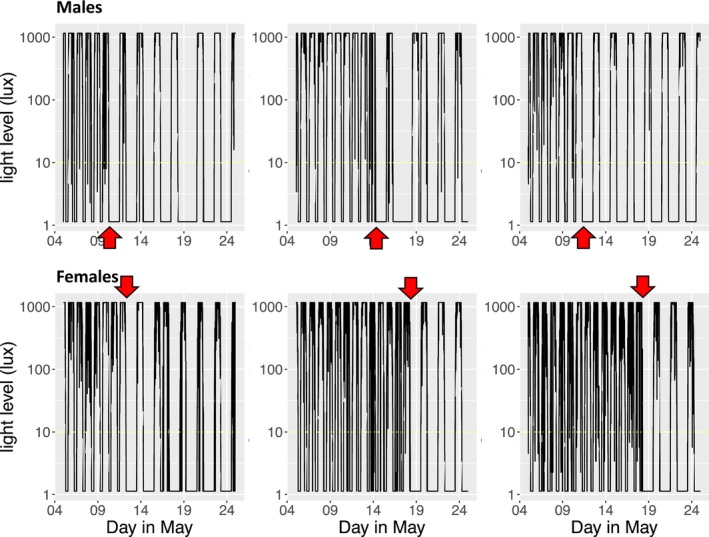
Representative examples of the raw light level waveforms from geolocation loggers with red arrows indicating the first dates of burrow re‐occupancy (start of incubation) for three male (top row) and three female (bottom row) rhinoceros auklets (*Cerorhinca monocerata*). These examples are from Middleton Island (Figure 1) in 2015. Individuals are not male–female mating pairs.

**FIGURE C1:**
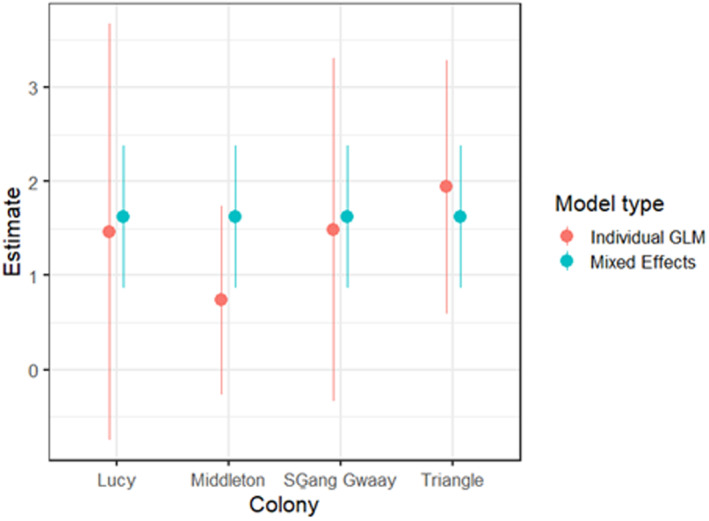
Model comparing estimates derived from individual general linear model (red) and linear mixed model (blue) fit to data from four different breeding colonies.

## Data Availability

The datasets analyzed during the current study are available in the Movebank Data Repository, https://doi.org/10.5441/001/1.618 (Hipfner et al., 2024).

## Appendix 1

### Additional Information on Location Uncertainty

The centroid for each wintering area represents the mean location derived from ~50 daily geolocations from January to February. Since these means represent a large sample, we can confidently expect that we have accurate location estimates for where birds spent the winter. Further, all the tags were analyzed in the same way, and so differences among tags were derived directly from the light records themselves. We do expect errors to exist in the absolute geolocations; however, the relative differences between the tags allowed us to test for correlations between laying date and distance measures.

We conducted a post hoc analysis to examine the sensitivity of the main result that depended on the geolocations. That is, birds that spent the winter further from their breeding colony had later breeding dates. We added noise to the distance measure by drawing a random value that had a mean of 0 and standard deviation calculated as a fraction of the observed distance, for example, new distance = observed distance + rnorm(0, observed distance × fraction), using a sequence of fractions from 0.05 to 0.50. With these new distance measures, we refit the GLMM, collated the GLMM parameter values for distance, and then examined how the relationship between distance and laying date varied with the added noise (Figure A1). We predicted that if the correlation was very sensitive to errors in the geolocation, then any additional noise would result in a loss of significance or change in the direction of the correlation.

This sensitivity analysis indicated that the main conclusion of a positive correlation between laying date and distance to wintering centroid became tenuous at about 15%–20% additional noise. As expected, when more noise was added, the more the parameter value began to approach zero (the dashed line; Figure A1). Additionally, the probability of seeing a statistically significant parameter value (at *α* = 0.05) also declined; at about 25%–30% additional noise, more than half of correlations were no longer significant. However, even at 20% additional noise, all the permutations indicated a positive correlation. Therefore, we conclude that the correlation between the laying date and distance to centroid was robust to a moderate amount of uncertainty in the geolocation.

## Appendix 2

### Methodology for Determining Lay Date in Rhinoceros Auklets

For both lay date identification methods (light/dark and wet/dry), we looked for alternating patterns of burrow occupation and vacancy. For the light/dark method, incubation behavior is indicated by an interruption in the waveform pattern—specifically an absence of light during expected daylight hours. Figure 1B shows examples of typical 24‐h light–dark cycles to the left of the red arrow, and interrupted light–dark cycles (extended dark periods) to the right of the red arrow indicative of incubation behavior. Similarly, using the wet/dry sensors we can determine when the bird remains out of the water for significant periods (5 h or more). Since these birds spend most of their lives at sea with minimal time on land, we can combine these methods to infer when incubation duties begin and thus estimate laying date.

To illustrate the utility of GLS light/dark waveforms for determining the start of incubation, Figure B1 presents the raw GLS light waveforms from six representative auklets (3 males, 3 females), where a normal period of 24‐h diurnal light/dark fluctuations indicative of life outside a burrow shifts abruptly into 24–48 h total darkness followed by a similar period of normal light levels. The initiation of this pattern can be interpreted as the re‐occupation of burrows for breeding (indicated by red arrows in Figure B1).

## Appendix 3

### Individual General Mixed Model Versus Linear Mixed Model Statistical Analyses

Middleton (*n* = 13), Lucy (*n* = 17), Triangle (*n* = 26), and SGang Gwaay (*n* = 20) have sufficient sample sizes that allow us to test individual relationships migratory distance and lay date. At reviewers' request, we compared parameter estimates between four individual general linear model (GLM) analyses versus the linear mixed‐effects model (LME) (Figure C1). In all four cases, the individual GLM showed the same positive association, but with reduced statistical significance due to larger standard errors. This difference was anticipated due to the lower sample sizes and which can result in an increased probability of Type II error.
